# A Short Review on Miniaturized Biosensors for the Detection of Nucleic Acid Biomarkers

**DOI:** 10.3390/bios13030412

**Published:** 2023-03-22

**Authors:** Madhusudan B. Kulkarni, Narasimha H. Ayachit, Tejraj M. Aminabhavi

**Affiliations:** 1 School of Electronics and Communication Engineering, KLE Technological University, Vidyanagar, Hubballi 580023, Karnataka, India; 2 Medical Physics Department, Wisconsin Institutes for Medical Research, University of Wisconsin, Madison, WI 53705, USA; 3 School of Advanced Sciences, KLE Technological University, Hubballi 580031, Karnataka, India

**Keywords:** biosensors, deoxyribonucleic acid (DNA), biomarker, microfluidics, point-of-care-testing (POCT)

## Abstract

Even today, most biomarker testing is executed in centralized, dedicated laboratories using bulky instruments, automated analyzers, and increased analysis time and expenses. The development of miniaturized, faster, low-cost microdevices is immensely anticipated for substituting for these conventional laboratory-oriented assays and transferring diagnostic results directly onto the patient’s smartphone using a cloud server. Pioneering biosensor-based approaches might make it possible to test biomarkers with reliability in a decentralized setting, but there are still a number of issues and restrictions that must be resolved before the development and use of several biosensors for the proper understanding of the measured biomarkers of numerous bioanalytes such as DNA, RNA, urine, and blood. One of the most promising processes to address some of the issues relating to the growing demand for susceptible, quick, and affordable analysis techniques in medical diagnostics is the creation of biosensors. This article critically discusses a short review of biosensors used for detecting nucleic acid biomarkers, and their use in biomedical prognostics will be addressed while considering several essential characteristics.

## 1. Introduction

Biosensing techniques have become one of the hot topics of interest that are exponentially growing due to their excellent performance and high throughput [1]. Biosensors have several advantages, such as high throughput, sensitivity, selectivity, real-time detection, ease of use, affordability, speed, and a minimum requirement of reagents [2,3,4]. Generally, biosensors were executed by assimilating the exclusive specificity of genetic responses and the great compassion of corporeal sensors. Thus, there has been an extensive opportunity for solicitations for biological and biochemical sensing methods, as these are pervasive in diverse fields such as food safety, healthcare, clinical, agriculture, environment, and pharmaceutics [5,6,7,8]. A biosensor is an analytical electronic microdevice that produces an electric signal via a bioreceptor and targets molecular interactions. Herein, the primary working principle of a biosensor is to sense the biorecognition incident and translate it into a determinate signal in a procedure called electronic signal processing [9,10]. The essential components of biosensors comprise (i) a biorecognition system or bioreceptor proficient in sensing the bioanalyte, (ii) a transducer that transforms the biorecognition component into a quantifiable signal, and (iii) an electric signal that is processed and incorporated to concoct the signal and to display it on a smartphone in an eco-friendly manner. Usually, biosensors can be categorized based on the bioreceptor event or transducer engaged for several applications. Firstly, in context, the receptors contain the recognition system, such as cells, nucleic acids, proteins, antibodies, peptides, and enzymes. Secondly, concerning the sort of transducer, such as optical (reflection, luminescence, absorption, refraction, fluorescence, etc.), electrochemical (amperometric, potentiometric, conductometric, impedimetric, etc.), and mass-based (piezoelectric) [11,12,13,14,15,16]. Biosensors show a prodigious prospective for evolving analytical tools with limitless applications in diagnosing, averting, and treating pathogens, mostly by identifying the associated specific biomarkers. In addition, these are anticipated to contest the ASSURED criteria recognized by the World Health Organization (WHO); the elaboration is as follows: affordable, sensitive, specific, user-friendly, rapid and robust, equipment-free, and deliverable to end-users [17,18,19,20,21]. Biosensors are commonly used as point-of-care-testing (POCT) devices for biomedical applications that can detect proteins, peptides, oligonucleotides, hormones, metabolites, etc.; these samples can act as biomarkers that are allied with the disease status [22,23].

In recent times, portability has attained a wide range of significance due to its potential applicability to the POCT [24]. The biorecognition of virus status at the receptor has gained wide consideration from several scholars and scientists in the past few decades. Nucleic acids are among the most significant biomarkers and are often studied biomolecules due to their strong connection to gene expression. Even minor DNA sequence variations can lead to distinguished biomedical and clinical insinuations. For example, single nucleotide polymorphisms (SNPs) are alleged to disclose the inherent information of specific predispositions to viruses and the mixed reactions to treatment. Thus, DNA-based biosensors are essential to integrate quantification tactics to identify the existence of common profuse mutations [25,26,27].

Recently, microfluidics has been associated with progressive ideas, which include lab-on-a-chip (LoC) or micro-total-analysis systems (μTAS), that are being established hastily in most research fields due to their assorted prospects and are set to transform the clinical, biochemical, medicinal, biological, healthcare, environmental, food, and agriculture industries [28,29,30]. This technology has emerged in innovative and scientific investigations for numerous biomedical and clinical applications. It deals with science and technology that processes a minuscule volume of reaction samples (<10^−18^ L) using microchannels ranging from 10 to 100 µm [31,32]. This primarily started its endeavor in the execution of gas chromatographic devices in the mid-1950s; successively, great appliances were being examined to drive and regulate the fluid flow within microfluidic devices [33,34,35,36]. Generally, microfluidic technology relates to fluid mixing, unraveling, relocating, and handling in a small capillary. Haeberle et al. [37] described several microfluidic platforms designed for applications related to microfluidic-based devices. In addition, for delicate identification, these microfluidic-based platforms can be easily coupled and comprehended to brace with diverse biosensor detectors [38,39]. The critical advantage of microfluidic-based biosensors can be recognized in their competence to manage micro/nanoliters of solutions, providing an opportunity to study with a small sample volume for bioanalytical POCT devices [40,41]. Furthermore, by utilizing hydrodynamic services in microchannels with various cross-sections, inertial microfluidic-based systems manage particles, atoms, molecules, and cells passively. These factors have led to the microfluidics field advancing toward large-scale commercial production, playing a significant role in the field’s development, especially in the biomedical area for detecting various biomarkers. Further, a receptor binds the sample, and a transducer transforms the response into an electric signal that electrochemical sensors can determine. Here, the electrode acts as a transducer in electrochemical sensors [42,43,44].

Recently, the biosensor definition was selected by the IUPAC standard; nevertheless, a modern-time appropriate description of the biosensor was earlier expressed by Newman. It refers to a compact analytical device integrated with a bioreceptor component associated with a physiochemical transducer. Clark was the pioneer in the subject of biosensors, publishing a study on the oxygen electrode in 1956. The 1st generation of biosensors were electrocatalytic devices that integrated enzymes with transducers that translated them into electrical signals. In the 2nd generation, affinity biosensors took advantage of discrete biological elements such as receptors, antibodies, and nucleic acids. Further, next generation biosensors are all of these interactions; an affinity interaction, such as DNA–DNA, antibody, antigen, protein, or DNA binding, controls the obligatory between the target analyte and the restrained biomolecule on the transducing element [16,45,46]. The immobilized molecule provides the biosensor system’s specificity. Since then, a sizable number of biosensors with applications in medical diagnosis have been reported in the literature. Actually, this field uses 80% of commercially available biosensor-based devices. Usually, biosensors are employed with several transducing components, such as thermometric, optical, electrochemical, magnetic, and piezoelectric. In the past decades, DNA-based sensing has begun to be used in clinical diagnostics for practical purposes to sense the presence of pathogenic types that cause infections, recognize genetic polymorphisms, and detect fact mutations [47,48,49,50]. Table 1 describes the prominent biosensor companies and target analytes involved in the market.

**Figure 1:**
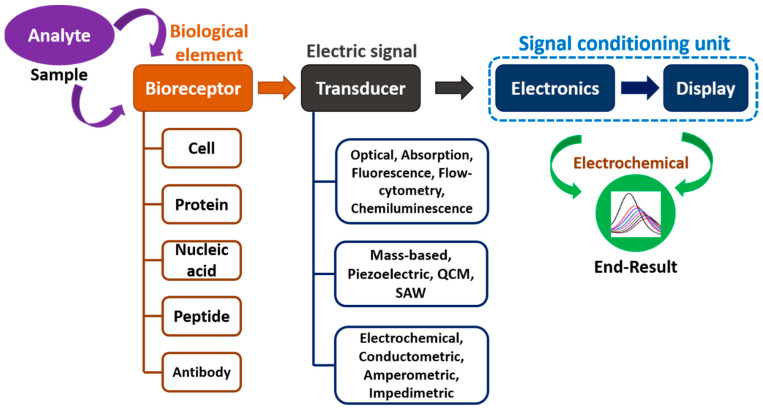
Block diagram representation of biosensors: from analyte to detection.

A biosensor is a logical apparatus that identifies deviations in biological progressions and translates them into electric signals. The term biological process can refer to any biomaterial such as tissues, acids, enzymes, cells, or microorganisms. It is an amalgamation of a biosensing component and a transducer, which transforms the information into electric indicators. Moreover, there will be an amplifier circuit that contains a signal conditioning platform, a controller or processor, and a display section. Figure 1 shows the block diagram of biosensors describing from the sample to the end-term detection.

## 2. Nucleic Acid-Based Biosensors

**Figure 2:**
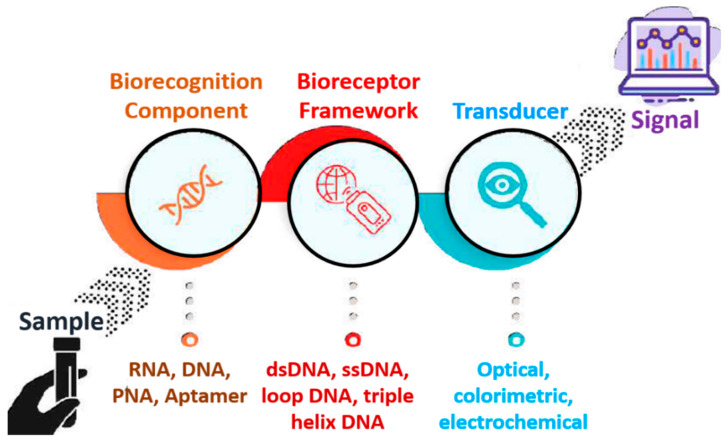
Classification of nucleic acid-based biosensors for detection of nucleic acid.

An analytical microsystem that includes an oligonucleotide with a known base sequence or composite construction of nucleic acid incorporated into or closely linked to a signal transducer is generally known as a nucleic acid biosensor [66]. DNA-based biosensors can be used to find biochemical or biological species and DNA/RNA templates. The majority of nucleic acid biosensors, also known as genosensors, are based on the extremely specific hybridization of complementary strands of DNA or RNA molecules [67,68]. The transducer is the element that transforms the biorecognition event into a quantifiable signal, while the probe serves as the biorecognition molecule and recognizes the target DNA when it is immobilized on the transducer surface. In nucleic acid biosensors, the hybridization event has been detected using a variety of detection technologies, including label-free ones such as piezoelectric and SPR transduction and others that frequently require labels, such as electrochemical approaches [69,70,71]. Recently, a number of reviews that explain all the critical facets of the transduction phase have appeared in the literature [72,73,74,75]. Figure 2 illustrates the classification of nucleic acid-based biosensors for the detection of nucleic acid.

The capture probe’s design is unquestionably the crucial preanalytical stage because the hybridization response’s specificity relies mainly on the bioreceptor element’s capabilities to seize oligonucleotides. The creation of linear probes uses a variety of commercially accessible firmware that may create detention of oligonucleotides inside highly conserved or hypervariable areas of various genomes following their assembly and alignment. Final tests include employing a basic local alignment search tool (BLAST) to check candidate sequences for homologies, theoretical melting temperature, dimer formation, and length (18–24 nucleotides) [76,77,78,79].

Stringency refers to the experimental factors influencing the hybridization occurrence at the transducer reaction-mixture interface, which typically comprise the composition of the hybridization and post-hybridization coating buffers and the response temperature. The fundamental prerequisite for a functional system when working with complicated sets of probes is the capacity of each probe to hybridize its target orders with high similarity and specificity under the same demanding circumstances. Additionally, some probes that can vary in chemical configuration and conformation have been used to assemble nucleic acid-based sensors [70]. Peptide nucleic acids (PNAs) are neutral N-(2-aminoethyl)-glycine pseudo-peptides that mimic DNA by attaching the nucleobases. PNAs have emerged as particularly intriguing oligonucleotide probe alternatives for the development of electrochemical sensing, primarily due to the vastly differing electrical properties of their molecular backbones [80,81,82,83]. There are many more articles that may be cited in support of this claim, but the authors believe that the works that have tested actual samples should be given more weight than those that have just been considered as a model sequence using less complex synthetic oligonucleotides [84,85].

**Figure 3:**
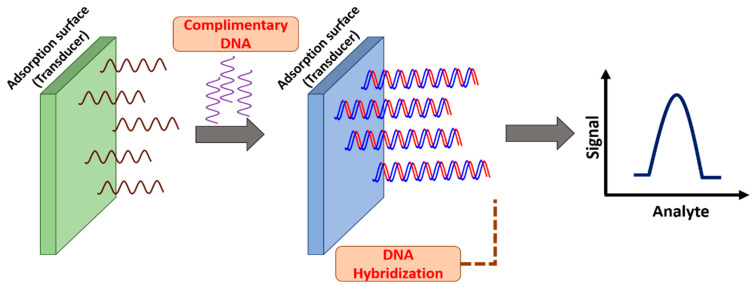
The bioreceptor component in nucleic acid-based biosensors for detecting nucleic acids resembles the hybridization process between DNA probe and target strands.

Because of their extensive utility in screening factors, which is crucial in medical diagnostics, drug delivery, and foodborne pathogens, these biosensors have recently gained relevance. DNA, peptide nucleic acid (PNA), RNA, and aptamers are the numerous kinds of DNA molecules [75,86]. This occurs when the target sequence complements the probe sequence immobilized on the transducer, exhibiting selectivity for non-complementary sequences. Additionally, the biosensor can be categorized according to the biorecognition component: Single-stranded DNA (ssDNA), also referred to as a DNA aptamer, double-stranded DNA, and triple-helical DNA are all examples of DNA-based biosensors. Figure 3 illustrates the hybridization process between the DNA probe and target strands [87,88].

### 2.1. Riboswitches

Riboswitches are mRNA components that regulate the production of mRNA on the same molecule in which they are encoded by binding to particular metabolite ligands. An expression platform and an aptamer domain make up riboswitches [89]. This switching in the secondary structure of the riboswitch regulates gene expression. Riboswitches are conceptually seen as an advancement of aptamer-based biosensing technology [90].

Small molecules such as ions, metabolites, or uncharged RNA can all be directly bound by riboswitches. A riboswitch in molecular biology is a regulatory section of a messenger RNA molecule that binds a tiny molecule, changing how the mRNA-encoded proteins are produced. Riboswitches can also recognize a variety of compounds with high specificity, including nucleic acids, peptides, carbohydrates, coenzymes, metallic ions, and amino acids [91]. Riboswitches are a promising alternative for biosensing methods since they can distinguish between molecules with identical structures [92]. Adenosylcobalamin concentrations have been effectively used as a biomarker of the metabolic phase in cell cultures, for instance, using a new biosensor that can distinguish between adenosylcobalamin and methylcobalamin.

### 2.2. Aptamers

These biosensors are a family of ssDNA or ssRNA oligonucleotides used in biochemistry. These are classified as nucleic acid-based biosensor biorecognition elements in biosensing systems. Targets that aptamers can bind to include proteins, medications, cells, and tissues. High selectivity and affinity have been reported for DNA aptamers, which bind to various analytes, including nucleic acids, cells, proteins, viruses, and tiny compounds such as aflatoxin B1, dopamine, cocaine, and metal ions. Additionally, it has been demonstrated that they can differentiate between enantiomers. However, due to the 2’-hydroxyl functional group in RNA aptamers, they often have a higher binding affinity than DNA aptamers to the identical target sequence [93,94].

The binding is the biorecognition event that promotes interaction between the target ligand and the aptamer, a modification in the conformation of the secondary and structures of the aptamer’s tertiary level. Consequently, in these structural modifications, aptamer-based biosensors are in charge of producing a frequently detectable signal. The antigen-antibody and aptamer identification models share certain resemblances regarding the binding pattern mechanism. A significant advantage of aptamers is their three-dimensional structure, which allows for high similarity and binding selectivity [95].

Aptamers are produced with excellent repeatability and inexpensive production costs, can withstand extreme ecological conditions, and can be kept without extra precautions. Finding the precise ssDNA/ssRNA sequences that can attach to the target ligand is the main obstacle in developing aptamer-based biosensors. As a result, libraries of random oligonucleotides and combinatorial approaches are used to select the aptamer sequence. It is a flexible tool since it enables the development of some variants to enhance properties such as the effectiveness and affinity of the aptamer [95].

### 2.3. Peptide Nucleic Acids (PNA)

The PNAs are synthetic DNA analogues that lack the sugar-phosphate backbone of natural nucleic acids in favor of a neutral peptide-like backbone made up of repeating N-(2-aminoethyl) glycine units. PNAs advantages over native nucleic acid substitute higher stability, including resistance to enzymatic cleavage, improved selectivity, neutral charge, and the ability to be synthesized using standard peptide solid-phase synthesis procedures are all features of DNA and RNA [96,97].

Environmental monitoring, food safety applications, and early disease diagnoses such as cancer have all benefited from the development of RNA or DNA detection biosensors based on PNA. Table 2 summarizes the performance characteristics of various nucleic acid biosensors.

The aforementioned biosensors’ performance traits, which include quick detection, competitive LOD, linear range, and specificity, are generally in line with the ASSURED standards. Most research strongly emphasizes parameter optimization, storage stability assessment, regeneration potential, manufacturing repeatability, and application to composite/genuine matrices [110]. It is essential to highlight the execution of nanomaterials for biosensing devices because they are crucial for cutting-edge biosensor designs and represent a rapidly growing field [111].

**Figure 4:**
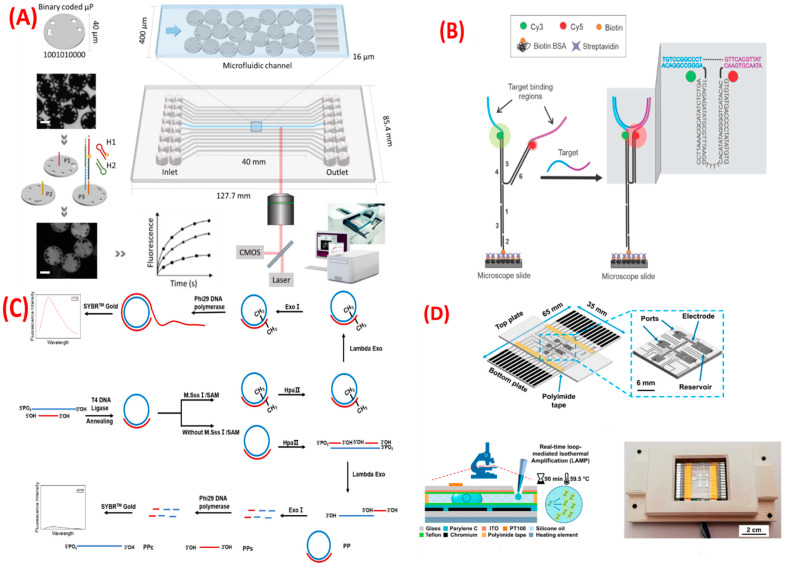
(**A**) Schematic representation of the components, including digitally barcoded nanoparticles, microfluidic cartridge, and an integrated instrument for the detection of molecular biomarkers [112] (**B**) Working principle of the nucleic acid-based nanotweezer sensor [113] (**C**) MTase activity uncovering by padlock probe with RCA technique [114] (**D**) Multi-view of the DMF device and accumulation [115].

Rutten et al. [112] demonstrated a fully integrated microfluidic platform encoded with nanoparticles combined with a microchannel for the simultaneous detection of several diseased-based biomarkers. Hybridization chain reaction (HCR) was investigated as a general strategy to achieve the anticipated sensibility by benefiting from this multiplex platform’s distinctive properties. The sensitivity was dramatically increased by an influence of 10^4^, down to low fM LOD values, compared to a non-amplified reference system (Figure 4A). The efficient but incredibly straightforward isothermal and protein-enzyme-free signal quantification technique achieves LODs for several targets ranging from 33 ± 4 to 151 ± 12 fM. Further, the proposed method can be used directly as a general strategy for the profound and accurate multiplex identification of diverse object compounds.

Anisa Kaur et al. [113] described a nanotweezers (NT)-based sensing approach that works on a single FRET pair and is proficient in sensing several objects. The authors proposed sensors that are sensitive to the low picomolar range (10 pM) and can distinguish between targets with a single base incongruity using nucleic acid mimics of miRNA biomarkers unique to triple-negative breast cancer (TNBC) (Figure 4B). These straightforward hybridization-based sensors have a lot of potential for sensitively detecting various nucleic acid biomarkers.

Wang et al. [114] designed a padlock probe (PP) to sense nucleic acid MTases, which syndicates target identification with the rolling circle amplification (RCA) technique without refinement or using other probes. By introducing MTase to the PP, which served as the enzyme’s substrate, the PP was methylated and protected against the cleavage reactions of HpaII, lambda exonuclease, and ExI, as well as absorption, and the PP started RCA. Thus, after mixing SYBR dye with the RCA, the fluorescent signal could be quickly identified (Figure 4C). This technique’s linear range for M.SssI was 0.5–110 U/mL, and the detection limit was around 0.0404 U/mL. Furthermore, tests for complex biological settings offer opportunities for potential use in complex ecosystems. The developed detection technique can additionally screen medications or inhibitors for MTases.

Coelho et al. [115] reported a digital microfluidics (DMF) technology made explicitly for LAMP of nucleic acid; real-time quantification was accomplished to screen the cancer biomarker c-Myc, which is linked to 45% of all human malignancies. Herein, 90 pg of the target nucleic acid (0.5 ng/L) was successfully amplified in less than an hour after completely modifying the sample and chemicals on this proposed platform. Additionally, using two mixing techniques that provide better fluorescence readouts, low-frequency alternating-current actuation and back-and-forth droplet motion onto the DMF droplets, we investigate the effectiveness of a novel mixing strategy in DMF (Figure 4D). The impacts of fluorophore bleaching are reduced through successive droplet irradiation and on-chip sample splitting via DMF procedures. Finally, compared to benchtop techniques, LAMP processes require 2 µL, a 10-fold volume reduction.

**Figure 5:**
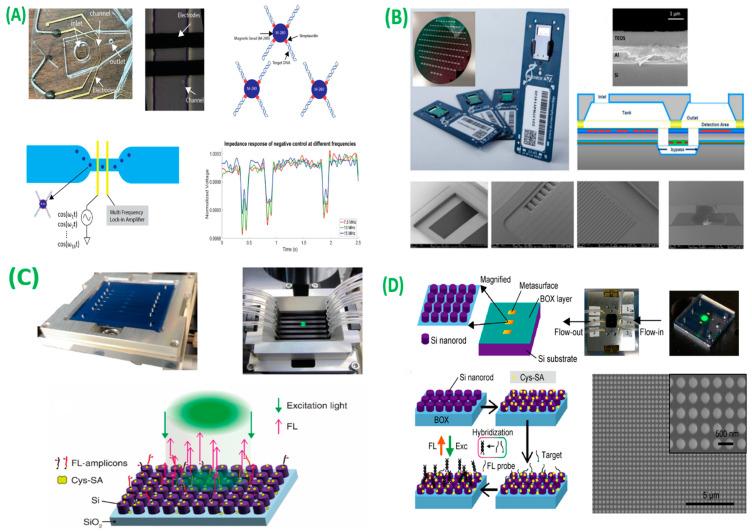
(**A**) Overview of the process and microscopic cross section of channel and electrodes [116] (**B**) Schematic of the LoC developed by STMicroelectronics [117] (**C**) Schematic of meta surface biosensors [118] (**D**) Schematic of dielectric meta surface structure and the whole view of meta surface substrate [119].

Kokabi et al. [116] proposed a neural network to prophesy nucleic acid quantities united to paramagnetic drops. To this end, a custom-built microfluidic channel was used to sense nucleic acid particles bound to slides by gauging the impedance peak response (IPR) at numerous occurrences. Here, electrical measurements comprise the event and imaginary/real portions of the highest concentration within a miniaturized device as the input of deep-learning simulations to forecast nucleic acid concentration (Figure 5A). Ten distinct deep-learning architectures were explicitly investigated. The proposed regression model’s results show that an R-squared of 96% and a slope of 0.66 are feasible.

Ventimiglia et al. [117] created a silicon lab-on-chip to detect nucleic acids using a hybridization microarray and integrated PCR. A couple of PCR microdevices with a volume capability of 11.2 L and a microarray-hybridization microchamber with a volume of 30 L, which are fluidically united by buried bypass and made up of silicon LoC, were created using bio-MEMS technology (Figure 5B). The nucleic acid microarray probe density was observed, which ranged from 1315 to 2075 probe µm^2^, and the LOD was 18 target µm^−2^. The direct identification of the beta-globin gene in human blood proved the principle for silicon microchips.

Iwanaga et al. [118] reported that a meta surface biosensor made up of an all-dielectric meta surface and a microfluidic transparent chip is described as a fast identification method that chains DNA magnification and a precise fluorescence biosensor. The meta surface biosensors recognized amplicons coming from attomolar SARS-CoV-2 nucleic acids, and the magnification was carried out within an hour, according to a series of proof-of-principle experimental results executed in the present scheme (Figure 5C). Furthermore, the dependable infection test criterion of 110 ribonucleic acid copies/150 L, which is a need, is significantly met by this detection competence.

Iwanaga et al. [119] described high-throughput and high-sensitivity meta surface fluorescence biosensors pertinent for DNA templates. The silicon-on-insulator nanorod array used in the all-dielectric meta surface biosensors has durable electromagnetic reverberations that recover fluorescence emission (Figure 5D). The meta surface fluorescence biosensors function well as an uninterrupted detection method.

## 3. Conclusions and Outlook

Over the past two decades, nucleic acid-based biosensors using microfluidic technology have shown progressive trends in healthcare applications with rapid, robust, reliable, and reusable advantages. Several designs of nucleic acid biosensing devices have been projected to record the reactions of discrete molecular biomarkers comprising lipids, tissues, cells, proteins, nucleic acids, and other molecules engaged in the bioprocesses. Modern nanotechnology, innovative enzyme engineering, exquisite designs based on complex sequence programs, precise base changes, and in vivo/in vitro applications of DNA-based biosensors have all facilitated their use. These days, a wide range of well-established nucleic acid-based biosensing devices have been described and extensively used in several fields, including food safety, ecological screening, disease prognosis, clinical prognosis, etc. Biosensors’ future endeavors can be divided into two categories: making them more specialized or generic; (i) A biosensor with a universal design is more suited for regular usage by regular people, even at home. Folks typically want a conclusion that is straightforward and suitable. The inability to implement a biosensor internationally is due to its lengthy and challenging operation, high cost, and instrument reliance. For instance, when attempting to validate the presence of SARS-COV-2, lateral stream assessment is unquestionably more feasible for a worldwide examination than RT-qPCR. (ii) Most likely, biologists, not chemists, are the ones who want a more consistent biosensor. An ideal biosensor could distribute precise information to track the presence of a bioprocess or realize its workings. Therefore, producing high-performance biosensors in living cells or organisms is critical. In spite of the substantial advantages of miniaturized biosensors in most of the domains, there is still a need for portable devices because of their exploration of space and control of machinery and processes. However, limitations of biosensors depicted in laboratory-based studies and the implementation of cutting-edge technology include design, development, stability, the lack of real-time data analysis amenities such as Bluetooth, internet-of-things (IoT), and specific challenges in cyber-physical-systems (CPS) and machine learning. Furthermore, these devices are still not fully active and reveal unconventional features that may widely apply to point-of-care testing and industrial-scale applications. To overcome the aforementioned challenges, a realization of real-time restraint hinders miniaturized biosensors from practically commercializing these with adaptability and flexibility. Moreover, a straightforward method for signal detection has been made available by the development of improved instrumentation. Therefore, developing advanced biosensors that integrate DNA nanotechnology with attractive equipment will be a promising future direction.

## Figures and Tables

**Table 1:** Prominent biosensor companies and targets involved in the market.

Company	Foundation Year	Targets	Cost (~$)	Ref.
Abbott	1888	Glucose biosensor	48.85	[51]
Affymetrix	1992	Pharmaceutical research	-	[52]
Applied Biosystems	1981	Affinity chip	69.95	[53]
ARKRAY, Inc.	1960	Creatinine biosensor	21.25	[54]
Bayer Diagnostics	1958	Glucose biosensor	42.78	[55]
Becton Dickinson	1897	Glucose biosensor	45.37	[56]
Biacore	1984	Affinity sensors for medical research	56.32	[57]
Cleome Innovations	2021	Medical POCT devices	18.26	[58]
Eppendorf Inc.	1989	Medical diagnostics	19.37	[59]
LifeScan	1981	Lactate	14.31	[60]
Molecular devices	1983	Pharmaceutical	-	[61]
Nanogen	1993	Glucose, urea, creatinine, and lactate biosensors	20.35	[62]
Roche Diagnostics	1896	Glucose biosensors	38.21	[63]
Renalyx Healthcare Systems	2013	Creatinine and albumin sensors	19.21	[64]
YSI Inc.	1948	Lactate	13.24	[65]

**Table 2:** Summary of performance characteristics for the various nucleic acid biosensors.

Biorecognition Component	Bioanalyte	Transducer	Limit of Detection	Application	Ref.
DNA	Nuclei acids	QCM	450 fM	Eco-friendly biointerfaces	[98]
ssDNA	RNA	Fluorescence	180 pM	Early disease diagnosis	[99]
PNA	ssDNA	GCE	2.58 pM	NS	[100]
PNA	miRNA-492 suggested biomarker for PDCA	Graphite oxide with gold NPs	8 nM	Early identification of PDCA	[101]
PNA	RNA	G-FET	0.2 aM	NS	[102]
Aptamer	PDGF	CNT	-	Atherosclerosis, fibrosis, malevolent viruses	[103]
ssDNA	Synthetic DNA of E. *faecalis*	Electrode with Gold NPs	3.4 amol L^−1^	Early detection of pathogens in food	[104]
Aptamer	Synthetic DNA of Group B *Streptococci*	Gold NPs	0.5 fM	Early detection of bacteria	[105]
Arched probeaptamer, Hairpin probe-1 and 2	*Salmonella typhimurium*	Luminescence	1.6 CFU mL^−1^	Adulteration in milk	[106]
3D-Printed PMMA chip	*Salmonella enteritis* and *Staphylococcus aureus*	Luminescence	5 CFU mL^−1^	Microfluidic-based biosensor for identification of virulent	[107]
Polyacrylamidehydrogel:aptamer strands	Microcystin-LR	Colorimetric	3.5 ng L^−1^	Detection of fresh fish	[108]
Aptamer hairpin	*Salmonella typhimurium*	Gold electrode	0.98 fM	Genomic DNA from clinical anal/vaginal samples	[109]

ssDNA = single-stranded deoxyribonucleic acid; PDCA = pancreatic ductal adenocarcinoma; PNA = peptide nucleic acid; RNA = ribonucleic acid; NPs = nanoparticles; GCE = glassy carbon electrode; QCM = quartz crystal microbalance; G-FET = graphene field effect transistors; CNT = carbon nanotube; PDGF = platelet-derived growth factor; NS: not specified.

## Data Availability

Not applicable.
